# Endocrinological complications of Duchenne muscular dystrophy and their subjective burden: observational study evaluating growth, puberty, and bone health

**DOI:** 10.1007/s40618-025-02699-x

**Published:** 2025-09-16

**Authors:** Marie Rohlenova, Jana Haberlova, Petra Holotova, Marketa Kumhera, Lucie Simkova, Barbora Lauerova, Iveta Svabova, Ondrej Soucek

**Affiliations:** 1 Department of Pediatric Neurology, Second Faculty of Medicine, Charles University and Motol University Hospital, Prague, 150 06 V Úvalu 84 Czech Republic; 2 Department of Rehabilitation and Sports Medicine, Charles University and Motol University Hospital, Prague, 150 06 V Úvalu 84 Czech Republic; 3 Department of Pediatrics, Second Faculty of Medicine, Charles University and Motol University Hospital, Prague, 150 06 V Úvalu 84 Czech Republic

**Keywords:** Duchenne muscular dystrophy, Fractures, Osteoporosis, Short stature, Delayed puberty, Questionnaire

## Abstract

**Purpose:**

Duchenne muscular dystrophy (DMD) and its treatment by glucocorticoids are associated with secondary osteoporosis, short stature, and delayed puberty. We aimed at exploring the prevalence and subjective burden of these endocrinological complications after the implementation of recommended care protocols.

**Methods:**

A prospective cross-sectional medical report review of boys with DMD followed at the largest pediatric neuromuscular reference center. An online questionnaire survey was part of the study.

**Results:**

We included 35 boys with DMD, aged 5.7–19.7 years, most of them on long-term daily glucocorticoid therapy (91%). Vertebral fractures were present in 50% of boys, short stature in 74%, and pubertal delay in 56% of boys. The glucocorticoid treatment duration and cumulative doses were associated with a higher prevalence of short stature, but not with the presence of vertebral fractures or pubertal delay. Areal bone mineral density assessed by densitometry only poorly identified patients with osteoporosis, compared to clear evidence of vertebral fractures by lateral spine radiograph. The boys were most concerned about the risk of fractures. Those in the pubertal age, however, were troubled also by their childish looks. The boys tolerated the surveillance protocols and treatment of complications very well.

**Conclusions:**

Vertebral fractures, short stature, and delayed puberty are very frequent among glucocorticoid-treated boys with DMD. Lateral spine radiograph is a crucial means for bone health assessment, with an even larger yield than densitometry. A questionnaire survey identifies patient needs and improves complex health care.

**Supplementary Information:**

The online version contains supplementary material available at 10.1007/s40618-025-02699-x.

## Highlights

Vertebral fractures, short stature, and delayed puberty are common in DMD treated by glucocorticoids.

Lateral spine radiograph is an irreplaceable tool for the detection of vertebral fractures, i.e., osteoporosis.

Among all endocrinological complications, fractures are of utmost concern in boys with DMD.

Patient questionnaire survey helps to identify patient needs and improve health care.

**Supplementary Information:**

The online version contains supplementary material available at 10.1007/s40618-025-02699-x.

## Introduction

Duchenne muscular dystrophy (DMD), an X-linked disorder caused by the lack of dystrophin, affects about 1 in 5000 boys [1]. Currently, there is no causal treatment, but the long-term use of corticosteroids (either prednisolone or deflazacort) has significantly improved the natural course of the disease [2–4], delaying the loss of ambulation by 1–3 years [2, 5], and combined with the implementation of ventilatory support and multidisciplinary care, the life expectancy has increased by approximately 10 years [2]. However, long-term glucocorticoid therapy has adverse effects, i.e. the aggravation of secondary osteoporosis, growth impairment, and delayed puberty.

Secondary osteoporosis in DMD is a common complication leading to low-trauma long bone and vertebral fractures [6, 7]. One of the most important factors leading to osteoporosis in DMD is the decrease or lack of weight-bearing activity due to muscular weakness [6, 8]. In addition, delayed puberty and long-term glucocorticoid treatment further aggravate bone loss [7–12]. The incidence of vertebral fractures in DMD hugely varies among studies (8–76%), depending on glucocorticoid type and dosing [7, 9, 13, 14] or whether lateral spine radiographs were systematically used to exclude asymptomatic vertebral fractures [7, 9, 15–19]. Short stature, defined as a height Z-score below − 2.0, is a well-known complication of DMD [20–24] and is among the negatively perceived complications of the disease by the boys themselves [20, 25]. Growth failure in boys with DMD was described already in steroid-naive infants and toddlers [21–23, 26, 27], and some of the dystrophin variants are associated with short stature [10, 24]. Long-term glucocorticoid treatment further worsens the growth of boys with DMD in a dose-dependent manner [10]. This may be attributed to local apoptotic effects of glucocorticoids in the growth plate [28], growth hormone (GH) resistance [29], and decreased GH secretion [30]. Besides osteoporosis and growth failure, long-term glucocorticoid treatment in boys with DMD also leads to delayed puberty [2, 4, 31] due to secondary hypogonadotropic hypogonadism [31–33]. The clinical consequences are further deterioration of growth, decline of aBMD, and also psychological distress due to marked physical differences from peers [25, 33].

The aims of the present study were, firstly, to observe the incidence and risk factors of the above-mentioned endocrinological complications in boys with DMD followed at a university referral tertiary care center, and secondly, to assess through a structured questionnaire the subjective feelings of the boys related to the endocrinological complications.

## Methods

### Patients and examinations

This was a cross-sectional observational study conducted in the years 2020–2021 in a single university neuromuscular center. The study was authorized by the Ethics Committee of Motol University Hospital in Prague (ref. nr. EK 912/19), and all the participants (or their parents) signed written informed consent. The study was conducted in accordance with the Declaration of Helsinki.

We enrolled patients with clinical features and genetically confirmed diagnosis of DMD aged 2–18 years, who were willing to participate. From the medical records, we retrieved data about the genetics, age at commencement, type, and dose of glucocorticoid treatment, age at loss of ambulation, site and age of fracture occurence, including the age of the detection of first vertebral fracture, presence, type and dose of endocrinological treatments, and longitudinal measurements of height and DXA LS aBMD Z-scores. The cumulative glucocorticoid dose was calculated after converting deflazacort to prednisolone equivalent by using the factor 2/3 [34].

In the routine outpatient visit, the actual height (cm) and weight (kg) were measured in the boys, and BMI (kg/m^2^) was calculated. We also gained parental heights. In non-ambulatory boys, the arm span was measured as a substitute for body height. The target height (TH) was calculated using the formula: TH = (mother’s height + father‘s height + 13)/2 [35]. Both height and TH were transformed to standard deviation scores (Z-scores) using the freely available software Rust.cz [36], designed for the Czech population based on the regular nationwide auxological survey [37]. The height reduction was calculated by subtracting the actual height Z-score from the TH Z-score.

Patients also underwent lateral imaging of the thoracic and lumbar spine, dual-energy x-ray absorptiometry (DXA) scans of the lumbar spine (LS) or of the whole body, and peripheral quantitative computerized tomography (pQCT) scanning of the forearm. Areal bone mineral density (aBMD) was conducted on Hologics HORIZON A device, and volumetric BMD (vBMD) was assessed by Stratec 2000 device. Images were evaluated by radiologists and reviewed by O.S. The age- and sex-specific, and height- and sex-specific Z-scores were automatically calculated by the original software of both devices. Trabecular vBMD at the metaphysis and cortical vBMD, polar strength-strain index, and muscle cross-sectional area at the diaphysis were assessed by pQCT. The detailed assessment protocol was described previously [38].

North Star Ambulatory Assessment (NSAA) was done in ambulatory patients who were able to perform the test. The pubertal assessment was done in boys older than 12 years, in order to detect boys who are at risk of delayed puberty and offer them a timely treatment.

Peripheral blood was drawn. All biochemical analyses were done in the same laboratory according to good clinical practice and standard lab protocols, which are available on the official website of Motol University Hospital [39]. Markers of bone metabolism (intact parathormone, 25-hydroxyvitamin D, calcium, phosphate, and alkaline phosphatase) and pubertal hormones in boys older than 12 years (folliculi-stimulating hormone - FSH, luteinizing hormone - LH, testosterone) were measured. In case of any pathological findings, the patients were referred to an endocrinologist (O.S.).

Based on the ISCD 2013 Pediatric Official Positions [40], boys fulfilling the criteria for secondary osteoporosis were treated with zoledronic acid i.v. dose 0.05 mg/kg every 6 months. The treatment was also indicated in boys after the loss of ambulation, with delayed puberty and a decrease in LS aBMD Z-score by more than 0.5 within a year, who were considered to have a high risk of fractures. We continued with the therapy until adulthood.

Based on the latest DMD care considerations, treatment by testosterone analogs was initiated in those older than 14 years with confirmed hypogonadism, and was considered in those older than 12 years on glucocorticoid treatment with no signs of pubertal development (defined as testicular volume below 4 ml, corresponding to Tanner stage 1 for genital development = G1) [4].

Pubertal induction was done by a mix of four testosterone esters administered i.m. every four weeks (starting dose of 50 mg was gradually increased by 50 mg every 6 months). We have adopted the protocol from [41], which was easy to follow and with a published end effect. By following this scheme, full adult dose (i.e. 250 mg) and full adult phenotype were usually achieved after 2.5 years. Patients continued the substitution beyond this time point with 250 mg every 4 weeks. None of the boys was treated with recombinant human growth hormone. If there were signs of both delayed puberty and secondary osteoporosis, the boys were treated both by ZA and Testosterone analogues.

### Questionnaire

All patients were asked to give their subjective perception of the presence of the endocrinological complications, the difficulties connected with adhering to the screening program, the treatment and its effects, as well as the side effects. This was done in April 2021 through an online structured questionnaire using the Survio.com website [42]. The boys were encouraged to fill in the forms themselves, but some forms were answered for the boys by their caretaker, depending on the age and abilities of the boys. The survey included only predefined answers, single or multiple choice. The translated version of the survey is available in the supplement.

### Statistical analysis

The statistical analyses were performed in the statistical computing environment R [43]. Continuous variables were described by the number of patients (in whom the measure was available), mean and standard deviation, and median, minimum, and maximum, where appropriate. The categorical variables were summarized by the number and percentage of patients in each category. Differences between the groups were tested at the 5% significance level, the test was chosen according to data distribution. Continuous data were tested by two-sided T-test or by the Wilcoxon Rank Sum Test (Mann-Whitney U Test) for non-normally distributed data. Categorical data were tested by Pearson’s chi-squared test or by Fisher´s exact test (where frequency < 5 was present). A log-rank test was used to evaluate the probability of low mineral density (DXA LS aBMD Z-Score below − 2.0) and the first detection of vertebral fracture in those with and without puberty. We used the age of first detection of these findings (gained retrospectively from patient charts) and the evaluation at the study visit as the pubertal status. A multiple regression analysis with forward selection, Cox model, was performed to test the dependence of age of VF detection and decrease of DXA below − 2.0 on glucocorticoid treatment duration, cumulative dose, ambulatory status, age at loss of ambulation, pubertal status, vitamin D concentration, and zoledronate treatment.

## Results

Thirty-five boys participated in the study. Table 1 summarises the main clinical characteristics of the cohort.

**Table 1:** Characteristics of the cohort

	*N*	mean (sd)	median (min; max)
Age (years)	35	12.5 (3.4)	11.8 (5.7; 19.7)
Height (Z-score)	35	−2.9 (1.4)***	−2.7 (−5.7; 0,7)
Height deficit* (Z-score)	34	−2.5 (1.5)***	−2.3 (−5.7; 1.1)
BMI (Z-score)	34	1.0 (1.8)**	1.4 (−4.7; 3.4)
GC treatment duration (days)	32	1901 (932)	1700 (391; 3938)
Cumulative GC dose (mg/kg)	32	945 (551)	886 (141; 3016)
Age at loss of ambulation (years)	18	10.7 (2.6)	10 (6; 16)
NSAA score	19	20.7 (9.6)	22 (2; 34)
LS aBMD (Z-score)	28	−1.9 (1.2)***	−1.9 (−4.4; 1.4)
TBLH BMC (Z-score)	18	−5.6 (2.2)***	−5.5 (−9.1; −2.3)
TBLH BMC (height-specific Z-score)	18	−3.3 (2.4)***	−2.2 (−7.7; −0.5)
TBLH aBMD (Z-score)	18	−5.7 (2.8)***	−5.6 (−12.2; −1.7)
TBLH aBMD (height-specific Z-score)	18	−3.0 (2.2)	−2.6 (−6.9; −0.3)
Trabecular vBMD (Z-score)	18	−1.9 (1.4)***	−1.8 (−3.9; 0.7)
Stress Strain Index (SSI) (Z-score)	18	−2.4 (0.7)***	−2.3 (−3.7; −1.2)

*Height deficit = Measured Height (Z-score) - Predicted Final Adult Height (Z-score). *BMI - body mass density*,* GC glucocorticoids*,* NSAA - North Star Ambulatory Assessment (expressed as count of Maximal 34)*,* LS aBMD - lumbar spine areal bone mineral density*,* Trabecular vBMD - Trabecular volumetric bone mineral density*

### Fractures and bone mineral density

There were seven long bone fractures in 6/35 boys (17%), and 17/34 (50%) boys suffered from at least one vertebral fracture. Long bone fractures were located at the femur (*n* = 2), tibia and/or fibula (*n* = 3), thallus (*n* = 1), and forearm (*n* = 1), and were caused by falls from the standing position (*n* = 3) or the sitting position in the wheelchair (*n* = 4). None of the detected vertebral fractures were preceded by apparent trauma. Glucocorticoid treatment did not differ between boys with and without vertebral fractures (Table 2). Also, the age at loss of ambulation, prevalence of the loss of ambulation, and NSAA score did not differ significantly between the two groups (Table 2). However, in the multiple regression model (including glucocorticoid treatment duration, cumulative dose, ambulatory status, age at loss of ambulation, pubertal status, vitamin D concentration, and zoledronate treatment as covariates), we found the age at loss of ambulation to be the best predictor for both the decrease of LS aBMD Z-score below − 2.0, and for the age at detection of first vertebral fracture (coef 0.69, *p* = 0.008; coef 0.77, *p* = 0.042, respectively).

**Table 2:** Comparison of values of potential risk factors between those with and without vertebral fracture (VF)

	VF yes (*N* = 17)	VF no (*N* = 17)	*p*-value
Age (years)	13.0 (2.7)	11.6 (3.8)	0.24
Height (Z-score)	−3.1 (1.4)	−2.9 (1.3)	0.68
Height deficit* (Z-score)	−2.8 (1.3)	−2.3 (1.5)	0.32
BMI (Z-score)	1.7 (1.3)	0.6 (1.7)	0.051
GC treatment (%)	16/17 (94%)	15/17 (88%)	1.0
GC treatment duration (days)	2224 (1016)	1604 (793)	0.068
Cumulative GC dose (mg/kg)	1125 (693)	790 (304)	0.093
Loss of ambulation (%)	10/17 (59%)	7/17 (41%)	0.49
Age at loss of ambulation (years)	10.3 (3.2)	10.9 (2.0)	0.67
NSAA score	17.3 (11.0)	23.3 (8.5)	0.22
LS aBMD (Z-score)	−1.6 (1.2)	−2.0 (1.0)	0.32
TBLH BMC (Z-score)	−6.1 (2.1)	−4.5 (2.3)	0.20
TBLH BMC (height-specific Z-score)	−3.5 (2.1)	−2.3 (2.5)	0.32
TBLH aBMD (Z-score)	−6.6 (3.0)	−4.1 (1.9)	0.055
TBLH aBMD (height-specific Z-score)	−3.2 (1.9)	−2.1 (2.1)	0.31
Trabecular vBMD (Z-score)	−2.2 (1.4)	−1.3 (1.5)	0.22
Stress Strain Index (SSI) (Z-score)	−2.4 (0.5)	−2.1 (0.9)	0.15
> 12 years and prepubertal (%)**	6/10 (60%)	1/6 (17%)	0.24

*Height deficit = Measured Height (Z-score) - Predicted Final Adult Height (Z-score), **Prepubertal = testicular volume < 4 ml (corresponding to Tanner stage G1). *BMI - body mass density*,* GC glucocorticoids*,* NSAA - North Star Ambulatory Assessment (expressed as count of Maximal 34)*,* LS aBMD - lumbar spine areal bone mineral density*,* Trabecular vBMD - Trabecular volumetric bone mineral density*

**Fig. 1:**
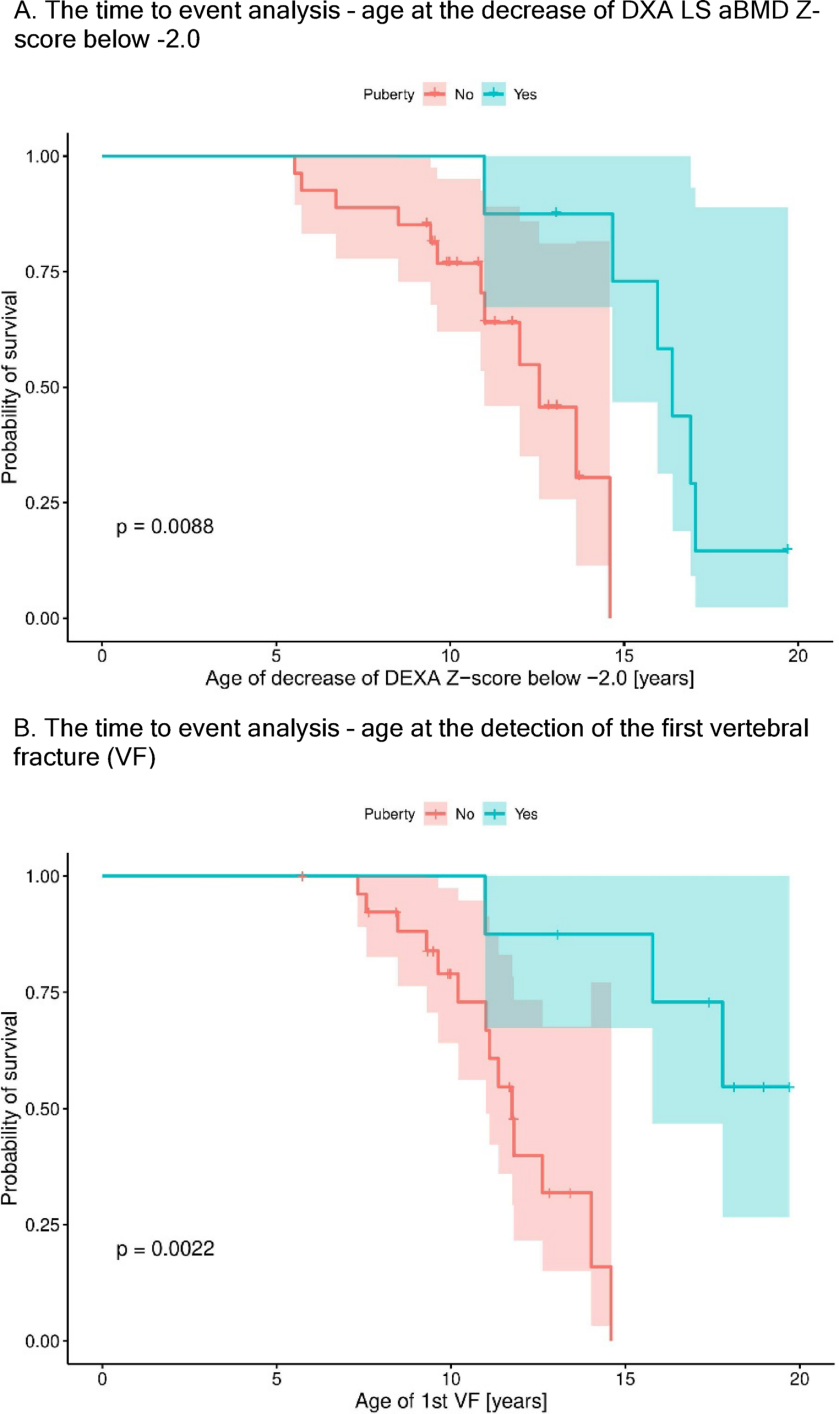
A Kaplan-Meier curve showing the risk of a decrease of DXA LS aBMD Z-score below − 2.0 (**A**), and the risk of detection of first vertebral fracture (VF) (**B**). The patients with puberty have 11.6 times lower risk of developing low bone mineral density of the lumbar spine, and 14.8 times lower risk of first vertebral fracture detection, at a given age. Log-rank test

Puberty was a protective factor, delaying the age of detection of first vertebral fracture, as well as delaying the decrease of LS aBMD Z-score below − 2.0 (Fig. 1). There were no significant differences in LS aBMD Z-scores, nor in height- or age-adjusted whole body aBMD and BMC Z-scores between those with and without vertebral fractures (Table 2).

DXA scans were completed in 28/35 (80%) boys. The whole body DXA scan was performed in 18/28, whereas 10/28 had DXA of lumbar spine only. Thirteen out of the 28 (46%) had low LS aBMD (Z-score ≤ −2.0). However, if short stature was considered, only two boys (7%) had low LS aBMD Z-scores. With regard to the height-specific whole-body Z-scores, 10/18 (55%) boys had low aBMC, and 9/18 (50%) had low aBMD. Table 3 shows, that the patients with normal and low LS aBMD did not differ in any of the other studied parameters. However, non-ambulant patients had lower LS aBMD Z-scores than the ambulant ones (−2.4 ± 1.0 and − 1.4 ± 1.3, *p* = 0.031). The presence of vertebral fracture was not associated with the LS aBMD Z-score, height-specific BMC Z-score, nor trabecular vBMD Z-score (linear regression, *p* = 0.203; 0.696; 0.096, respectively).

**Table 3:** Comparison of values of potential risk factors between those with low and normal areal bone mineral density (aBMD)

	LS aBMD Z-score ≤ −2.0 (*N* = 13)	LS aBMD Z-score > −2.0 and < 2.0 (*N* = 15)	*p*-value
Age (years)	13.5 (4.1)	11.4 (2.0)	0.11
Height (Z-score)	−3.3 (1.7)	−2.7 (1.3)	0.34
Height deficit* (Z-score)	−2.7 (1.6)	−2.6 (1.3)	0.86
BMI (Z-score)	0.6 (2.3)	1.7 (0.9)	0.14
GC treatment (%)	11/13 (85%)	15/15 (100%)	0.40
GC treatment duration (days)	2179 (1284)	1785 (710)	0.37
Cumulative GC dose (mg/kg)	1072 (837)	886 (369)	0.50
Loss of ambulation (%)	8/13 (62%)	5/15 (33%)	0.27
Age at loss of ambulation (years)	11.0 (3.0)	8.8 (1.1)	0.089
NSAA score	25.2 (6.1)	20.5 (10.8)	0.28
Trabecular vBMD (Z-score)	−1.8 (1.1)	−2.2 (1.5)	0.62
Stress Strain Index (SSI) (Z-score)	−2.7 (0.7)	−2.2 (0.7)	0.05
> 12 years and prepubertal (%)**	5/10 (50%)	2/4 (50%)	1.0

*Height deficit = Measured Height (Z-score) - Predicted Final Adult Height (Z-score), **Prepubertal = testicular volume < 4 ml (corresponding to Tanner stage G1). *BMI - body mass density*,* GC glucocorticoids*,* NSAA - North Star Ambulatory Assessment (expressed as count of Maximal 34)*,* LS aBMD - lumbar spine areal bone mineral density*,* Trabecular vBMD - Trabecular volumetric bone mineral density*

In our cohort, 17/35 (49%) boys were treated with zoledronic acid. The average age at initiation of treatment was 10.9 ± 2.4 years. There were no significant differences in serum concentrations of calcium (2.4 ± 0.12 vs. 2.5 ± 0.11, *p* = 0.063), parathormone (3.0 ± 1.6 vs. 3.5 ± 1.6, *p* = 0.33) or 25-OH-vitamin D (51 ± 14 vs. 44 ± 14, *p* = 0.16) between treated and untreated boys. 

### Short stature

The heights of the boys were lower than expected, as compared to both the healthy population and their target heights (Table 1). There were 74% (26/35) of boys with short stature, and 62% (21/34) two or more SDS shorter than their target height. The target height of one boy could not be calculated because of the unknown height of his biological father. Both the cumulative glucocorticoid dose and treatment duration were higher in boys with short stature as compared to boys with normal stature (Table 4).

**Table 4:** Comparison of values of potential risk factors between those with short and normal stature

	Height Z-score ≤ −2.0 (*N* = 26)	Height Z-score > −2.0 and < 2.0 (*N* = 9)	*p*-value
Age (years)	12.6 (3.2)	12.3 (4.5)	0.89
Height deficit* (Z-score)	−3.3 (1.1)	−1.1 (0.9)	< 0.001
BMI (Z-score)	1.0 (1.7)	0.9 (2.1)	0.88
GC treatment (%)	23/26 (88%)	9/9 (100%)	0.71^#^
GC treatment duration (days)	2127 (947)	1323 (695)	0.016
Cumulative GC dose (mg/kg)	1095 (569)	562 (304)	0.002
Loss of ambulation (%)	12/26 (46%)	6/9 (67%)	0.50^#^
Age at loss of ambulation (years)	10.7 (2.6)	10.8 (3.1)	0.91
NSAA score	21.7 (9.7)	17.0 (10.7)	0.46
LS aBMD (Z-score)	−2.0 (1.0)	−1.4 (1.8)	0.36
TBLH BMC (Z-score)	−5.6 (2.2)	−5.8 (2.5)	0.85
TBLH BMC (height-specific Z-score)	−2.9 (2.1)	−4.9 (3.1)	0.29
TBLH aBMD (Z-score)	−5.9 (2.9)	−5.2 (2.4)	0.64
TBLH aBMD (height-specific Z-score)	−2.6 (1.9)	−4.4 (2.9)	0.31
Trabecular vBMD (Z-score)	−1.7 (1.4)	−3.0 (1.3)	0.40
Stress Strain Index (SSI) (Z-score)	−2.4 (0.6)	−2.1 (1.2)	0.27
> 12 years and prepubertal (%)**	6/14 (43%)	1/3 (33%)	1.0^#^

*Height deficit = Measured Height (Z-score) - Predicted Final Adult Height (Z-score), **Prepubertal = testicular volume < 4 ml (corresponding to Tanner stage G1). *BMI - body mass density*,* GC glucocorticoids*,* NSAA - North Star Ambulatory Assessment (expressed as count of Maximal 34)*,* LS aBMD - lumbar spine areal bone mineral density*,* Trabecular vBMD - Trabecular volumetric bone mineral density*

### Puberty

**Fig. 2:**
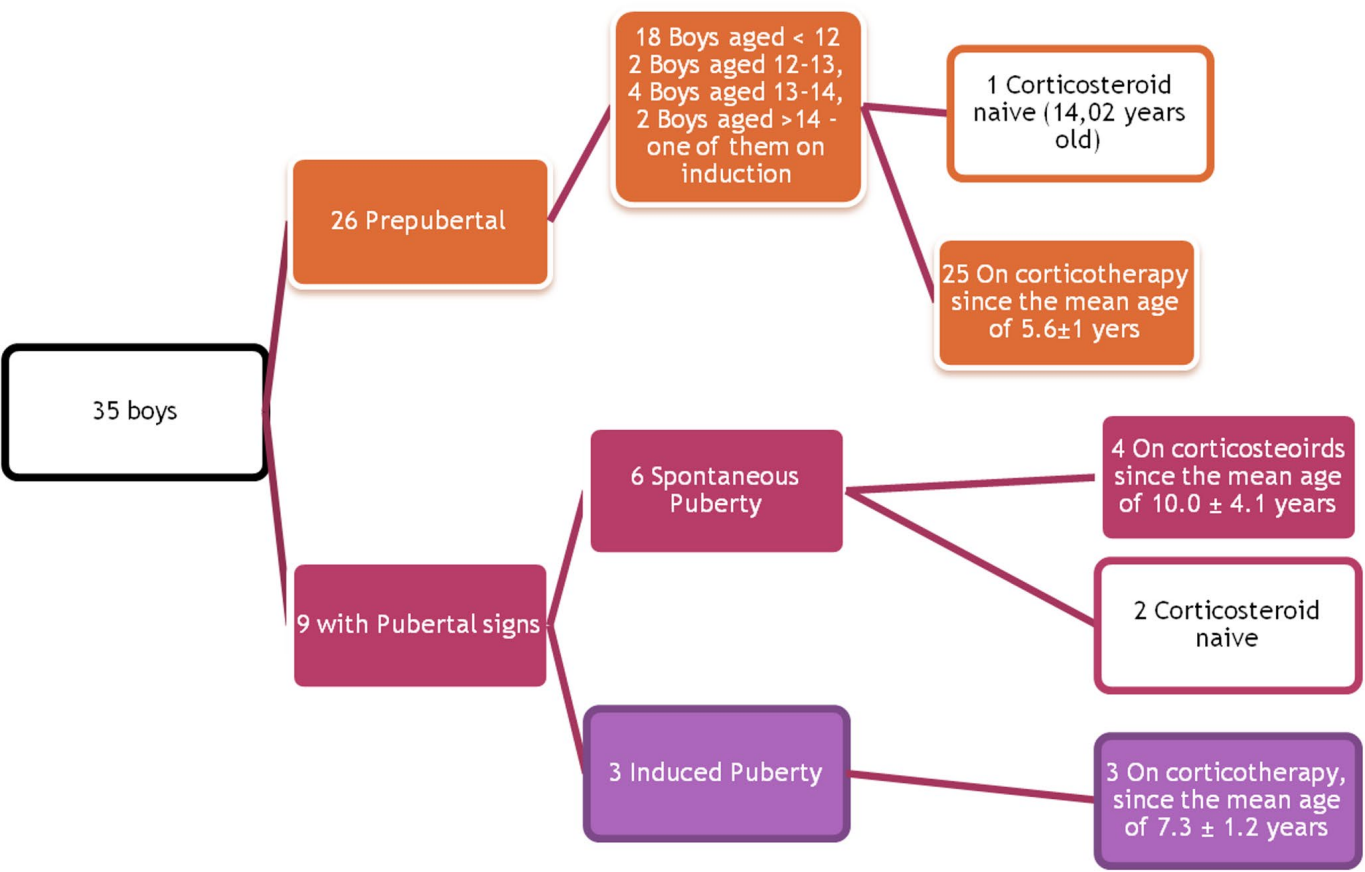
The diagram shows the proportion of prepubertal and pubertal boys in our cohort. Note that most boys were prepubertal. Of those, the Majority were younger than 12 years of age, Making it difficult to assess the true prevalence of delayed puberty in the cohort. Of those older than 14 years of age, delayed puberty was present in every second boy. Notably, the boys with spontaneous puberty tended to have corticosteroids initiated at a younger age than those with either pubertal induction or no signs of pubertal development after 14 years of age. However, the difference was not statistically significant, and there was one boy with no pubertal signs at 14 years, who never used corticosteroids

Of the 9 boys older than 14 years, 5 (56%) were either prepubertal (*n* = 2) or had had a pubertal induction for delayed puberty (*n* = 3). Figure 2 shows the proportion of boys with and without pubertal signs, and the age at initiation of glucocorticoid therapy in each group. From the cohort, 4 boys had had pharmacological induction of puberty (one of those did not have pubertal signs yet). All these 4 boys were also treated with zoledronate for secondary osteoporosis.

### Questionnaire

The questionnaire was filled in by 27/35 (77%) participants. The translated questions and results, as provided by Survio.com, can be found in the Supplement.

Among the three selected endocrinological complications, increased fracture risk (osteoporosis) was considered by far the most important (indicated by 19/27, i.e. 70% of respondents), followed by short stature (6/27; 22%) and delayed puberty (2/27, 7%). Regarding the overall health status, the most important desire (scored from 1 – the most important, to 5 – the least important) of the boys was to maintain gait and muscle strength in arms to perform routine daily activities (average score 1.70). This was followed by the desire for independence (average score 3.04), the same height as peers (score 3.52), reduction of fracture risk as much as possible (score 3.96), normal pubertal development (score 4.33), and shorter hospital stays (score 4.44). Back pain (sometimes weak and sometimes strong pain, and pain very often) was reported by 16/27 (59%) respondents, whereas no pain (or rarely) was present in 11 (41%). There was no association between back pain (sometimes strong pain and pain very often) and the presence of vertebral fracture (*p* = 1.0, the Fisher exact test). The questionnaire was filled in by 12/17 boys treated with zoledronate. Of those boys, 83% (10/12) reported back pain before the initiation of the treatment, but in 70% (7/10) boys, the pain was reduced after one year of zolendronate treatment.

Adverse effects after each administration were reported by 1/12 respondents (8%), only after the first application by 8/12 boys (67%), and never by 1/12 respondents (8%). Two participants (16%) chose not to answer this question. The most difficult aspect related to zoledronate administration was i.v. access (25%) or long-distance traveling (17%). However, 42% of respondents stated that the treatment brings them no discomfort. Short stature was (very or rather) distressing for 7/27 (26%) respondents, not (at all or rather not) distressing in 12/27 (44%), and 8/27 (30%) could not decide. Delayed puberty was found to be (very or rather) distressing in 5/27 (18%) respondents, not or rather not distressing by 11/27 (41%) respondents, and 11/27 (41%) boys could not decide. Participants who were worried about delayed puberty were all older than 12 years. The most common troubles (selected from a pre-specified list, multiple choice) associated with delayed puberty were the loss of autonomy in communication with strangers due to childish appearance (10/27, i.e. 37% of respondents), the physical aspect and interests distinct from the peers (9/27, i.e. 33%), and health issues such as low bone density (5/27, i.e. 19%). On the other hand, 8/27 (30%) boys did not mind at all. Interestingly, regular assessment of the pubertal stage in the neuromuscular outpatient clinic would be very or rather bothering for 6/27 (22%) respondents, 30% could not decide, and 48% would not mind (at all or rather not).

## Discussion

The main contribution of this study is the comprehensive description of the prevalence and subjective burden of endocrinological complications, including patient-reported outcomes, in a cohort of boys with DMD followed in the largest pediatric neuromuscular ERN center in the country, after the implementation of the latest DMD clinical surveillance protocols [4, 44]. We found out that: (1) every other boy with DMD had vertebral fracture, which is an important indicator of osteoporosis, (2) non-ambulant patients had lower LS aBMD Z-scores than the ambulant ones, and the age at loss of ambulation was the best predictor of age at first vertebral fracture detection and of low bone mineral density (3) 74% of boys had short stature, with significant association with the cumulative glucocorticoid dose and treatment duration, (4) pubertal delay affects half of the boys and (5) the most bothering complication was increased fracture risk and the biggest desire was to maintain gait and muscle strength in arms to perform routine daily activities.

### Bone health

The high prevalence of vertebral fractures in our cohort is in accordance with previous studies in pediatric DMD cohorts treated with corticosteroids, with the reported incidence ranging from 6 to 75% [7–10]. The prevalence of vertebral fractures in our study was up to 10 times higher than the prevalence reported in comparable retrospective cohorts [45, 46] in which no routine vertebral fracture screening was performed, suggesting a significant proportion of boys with DMD with asymptomatic vertebral fractures. According to the results from our questionnaire study, vertebral fractures were not associated with severe back pain. However, the answers might have been influenced by zoledronate treatment, as back pain relief was the most commonly reported positive effect of the therapy. Lateral spine x-ray performed at least every second year, especially in boys older than 10 years of age, and if treated with glucocorticoids, thus seems to be a justified routine assessment.

Contrary to our cohort, lower aBMD Z-scores have been previously associated with an increased risk of fractures in the general pediatric population [47] as well as in children with cerebral palsy and muscular dystrophy [48]. Moreover, there was a significantly lower aBMD in a retrospective study evaluating bone health in boys with DMD with multiple and more severe vertebral fractures, in comparison with boys with early stages of vertebral fractures detected by routine screening [19]. Another study of 252 patients with DMD showed that lumbar spine aBMD did not correlate with either motor functional scale or the risk of fractures, contrary to the whole body aBMD and femoral neck aBMD [8]. We have not observed such a correlation. These contradictory findings suggest that DXA assessment alone doesn’t sufficiently identify patients at risk and further support the need for routine lateral spine x-ray scanning in boys with DMD.

Our finding that ambulation is associated with higher aBMD in boys with DMD compared to non-ambulant boy, as well as the age at loss of ambulation being the best predictor for the age of detection of first vertebral fracture and occurence of low LS aBMD, is in agreement with previously published reports, such as in a study of 41 boys with DMD, where the aBMD, both of the lumbar spine and femur, was significantly lower in the non-ambulant boys than in the ambulant ones [13]. Furthermore, in a study of 292 ambulant glucocorticoid-treated boys, aBMD declined with the worsening of functional motor scale, the femoral aBMD being influenced sooner than whole body aBMD. Therefore, despite that the link between aBMD and fracture occurrence is not straightforward, ambulation status represents an important indicator of bone health in DMD, and the loss of ambulation in these patients should trigger a full bone health assessment.

### Short stature

Three-quarters of our boys had short stature, and there was an association with the cumulative glucocorticoid dose and treatment duration. This is in agreement with previous studies, where even glucocorticoid naive boys had an average height Z-score of −1.0 [21, 24], and in glucocorticoid-treated boys, the height was significantly lower (mean Z-score +-SD, p-value) than in the untreated boys [49]. According to the questionnaire, only one-quarter of the boys were concerned about being shorter than their peers. Contrary to our finding, small stature was a serious issue for nine out of ten boys from a UK survey study on boys with DMD treated for delayed puberty [25]. In our survey, boys with DMD deemed the other complications more important than impaired growth, similar to adult patients with DMD from the UK [33]. It seems obvious that subjective feelings change with the mental development of the individual. The differences between studies might therefore be caused by the various ages or pubertal stages of the respondents.

There is some evidence suggesting that short stature might be beneficial for the preservation of muscular function, as shorter muscle fibers are more resistant to mechanical loading [50, 51]. According to one retrospective study, shorter boys with DMD indeed maintained their gait longer than the taller ones [52]. In concert with that, we have previously shown on retrospective data that the risk of short stature was larger in ambulant boys than in wheelchair-bound boys of the same age [53]. However, in the present study, we did not observe any beneficial role of short stature on the preservation of muscular strength or gait. The size of our cohort, as well as the cross-sectional character of the study, might have prevented us from observing such an association. Further research is needed to elucidate whether short stature bears some muscle protective role in DMD.

### Puberty and its predictors

The proportion of delayed puberty was similar to previous studies [54, 55]. Delayed puberty has not been described in steroid-naïve boys with DMD [10], on the contrary, the early initiation of glucocorticoid therapy often leads to delayed puberty [12, 21, 56]. Our study was underpowered to perform such an analysis. While glucocorticoid treatment remains the main anticipated cause of delayed puberty in DMD, alternative treatment protocols (as compared to daily use) or novel glucocorticoid analogs (i.e., VBP-15/vamorolone) could prove to be less harmful to the natural pubertal development.

Normal pubertal development might be a protective factor for bone health, as suggested previously [32, 56]. Our data support these findings, stressing the importance of pubertal evaluation and timely treatment of delayed puberty in boys with DMD.

According to the questionnaire, delayed puberty distressed only one-fifth of the respondents (5/24), and all of them were older than 13 years. The Majority of respondents were younger than 13 years and did not have a clear opinion about pubertal development. This is contrary to the survey conducted in the UK [25], where the lack of pubertal development was distressing for most of the adult participants. The testosterone treatment was very well tolerated in that study. The most undesirable effects of absent puberty were very similar in our cohort and the British study - being considered younger, which is connected with decreased self-reliance, and looking different from peers. The risk of health consequences of delayed puberty was the least frequently reported fear in our questionnaire survey. Our findings highlight the need for patient age-specific education and care, in which pubertal delay and its consequences shall be a focus in patients older than 12–13 years.

### Surveillance and treatment of endocrinological complications

Endocrinological complications are an important issue for patients with DMD. The most disturbing complication was increased fracture risk, and the biggest desire of our boys with DMD was to maintain gait and muscle strength in their arms to perform routine daily activities. Interestingly, the additional time spent in the hospital due to the monitoring and treatment of complications (when compared to our previous routine care) was not inconvenient to the boys, and the families preferred being thoroughly monitored, to prevent complications. This underlines the importance of including patient perspectives to create the specialized care plan.

There was quite a large proportion of respondents who did not have a clear opinion about the issues that were studied. This might be due to either timidity while talking about delicate themes, the lack of complete understanding of some of the questions (given the online format of the survey with no possibility to ask further questions), or due to focusing on other disease complications and problems. This suggests that repeated and structured education is an inseparable part of multidisciplinary care for boys with DMD.

### Strengths and limitations

The strength of the study is a comprehensive assessment of endocrinological complications in boys with DMD followed in the largest pediatric reference center, and the complementary questionnaire survey of patients‘ perspectives. On the other hand, a relatively small cohort of patients may limit the results. In particular, we were not able to test whether zoledronate treatment contributed to the lack of association between aBMD and vertebral fractures or whether glucocorticoids cause pubertal delay.

## Supplementary Information

Below is the link to the electronic supplementary material.

Supplementary Material 1

## Abbreviations

DMD: Duchenne Muscular Dystrophy
SD: Standard Deviation
GH: Growth Hormone
NSAA: North Star Ambulatory Assessment
TH: Target Height
DXA: Dual Energy X–Ray Absorptiometry
aBMD: Areal Bone Mineral Density
pQCT: Peripheral Quantitative Computerized Tomography
vBMD: Volumetric Bone Mineral Density
