# Supplementary material for: Endocrinological complications of Duchenne muscular dystrophy and their subjective burden: observational study evaluating growth, puberty, and bone health
Source: J Endocrinol Invest. 2025 Sep 16;49(1):119–30. doi: 10.1007/s40618-025-02699-x (PMC12847132; doi:10.1007/s40618-025-02699-x)
Supplement: Supplementary file 1 — Supplementary Material 1 [file 40618_2025_2699_MOESM1_ESM.pdf]

Sledování endokrinologických komplikací a léčby u chlapců s DMD

Základní údaje

Název výzkumu	Sledování endokrinologických komplikací a léčby u chlapců s DMD
Autor	
Jazyk dotazníku	Čeština
Veřejná adresa dotazníku	https://www.survio.com/survey/d/U7C0H5U3E9U2G5J1T
První odpověď	29. 03. 2021
Poslední odpověď	17. 04. 2021
Doba trvání	19 dnů

Statistika respondentů

58	28	0	30	48,3%
Počet návštěv	Počet dokončených	Počet nedokončených	Pouze zobrazení	Celková úspěšnost vyplnění dotazníku

Historie návštěv (29. 03. 2021 – 17. 04. 2021)

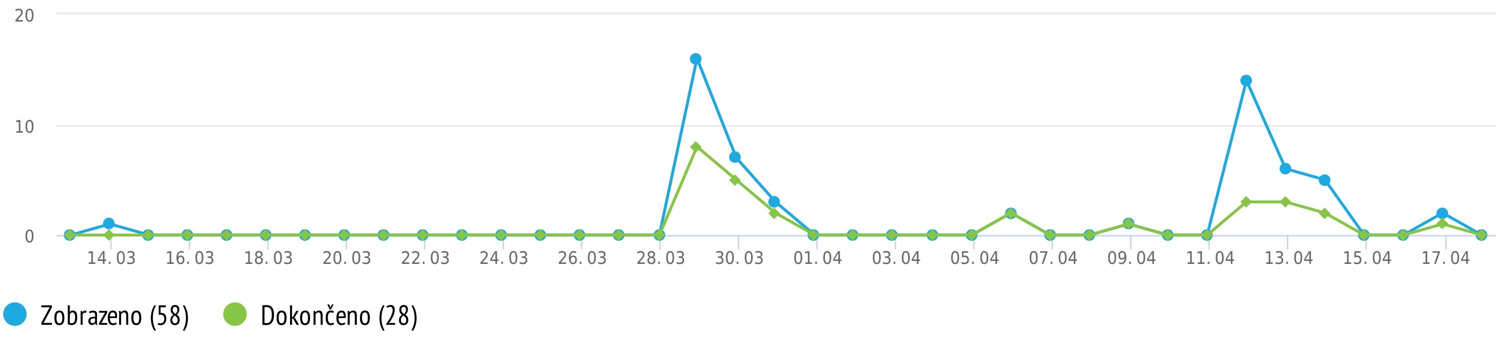

Celkem návštěv

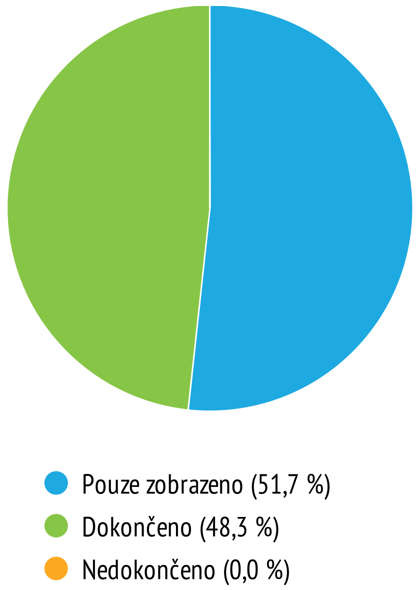

Zdroje návštěv

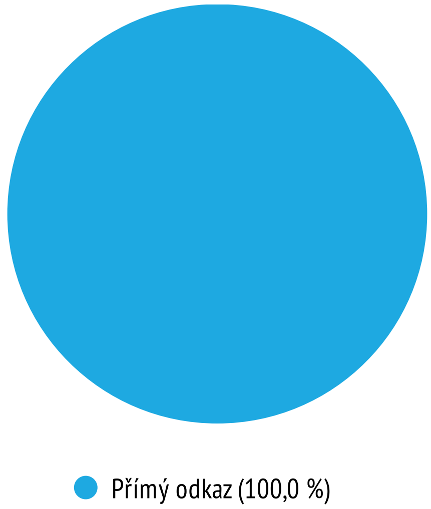

Čas vyplňování dotazníku

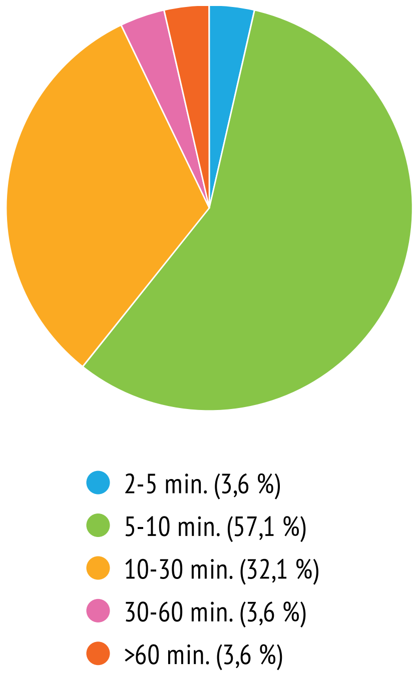

Filtry podle

⊗	*Vyřazených odpovědí*	1

Výsledky

1 Jaké jsou Tvoje iniciály? (Napiš prosím první písmeno ze svého jména a ze svého příjmení)

Textová odpověď, zodpovězeno 27 x, nezodpovězeno 0 x

- AE
- BF
- DH
- DP
- DT
- D.T
- Filip
- Hoai Nam Le
- JC
- Jiří Kucera
- J. K.
- JR
- J T
- JV
- KM
- LK
- MD
- MK
- MR
- ms
- OS
- PŠ
- RD
- TK
- T.L.
- Václav Veselý
- V.P

2 Kolik je ti let?

Textová odpověď, zodpovězeno 27 x, nezodpovězeno 0 x

- (4x) 10
- (3x) 11
- (3x) 12
- (3x) 13

- (4x) 14
- 15
- 17
- 18
- (2x) 19
- 20
- 7
- 8
- (2x) 9

3 Kdo odpovídá na otázky dotazníku?

Výběr z možností , zodpovězeno 27 x, nezodpovězeno 0 x

Možnosti odpovědí	Responzí	Podíl
Já sám za sebe (s případnou technickou dopomocí)	7	25,9%
Vyplňuje za mě rodič / opatrovník	20	74,1%

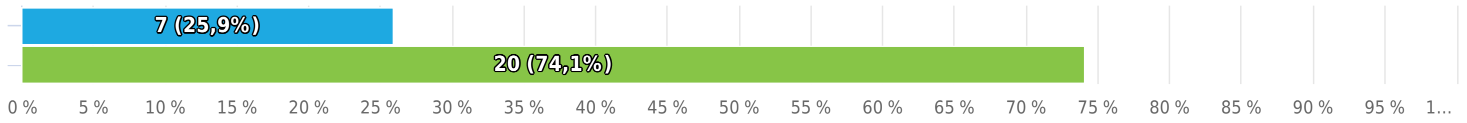

4 Užíváš kortikoidy?

Výběr z možností , zodpovězeno 27 x, nezodpovězeno 0 x

Možnosti odpovědí	Responzí	Podíl
Ano	24	88,9%
Ne	3	11,1%

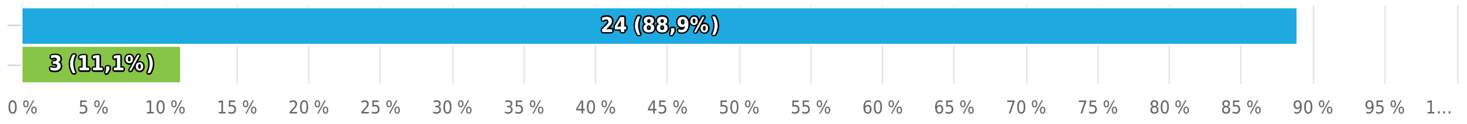

5 Měl jsi někdy nějakou zlomeninu?

Výběr z možností, více možných , zodpovězeno 27 x, nezodpovězeno 0 x

Možnosti odpovědí	Responzí	Podíl
Ano - ruky nebo nohy	5	18,5%
Ano - kompresivní zlomeninu obratlů (lékař to poznal dle RTG páteře)	5	18,5%
Ano - jinou	3	11,1%
Ne, nikdy jsem nic zlomeného neměl.	18	66,7%

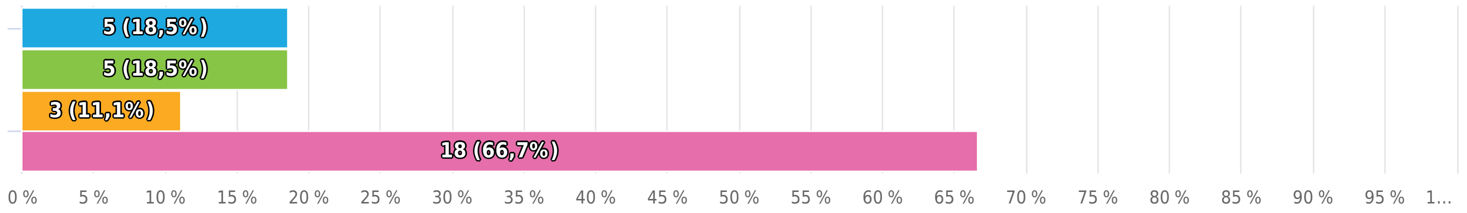

6 Trpíš na bolesti zad?

Výběr z možností , zodpovězeno 27 x, nezodpovězeno 0 x

Možnosti odpovědí	Responzí	Podíl
Ne nebo jen výjimečně	11	40,7%
Občas, bolest je mírná	11	40,7%
Občas, bolest je silná	2	7,4%
Velmi často	3	11,1%
Ano, každý den	0	0,0%

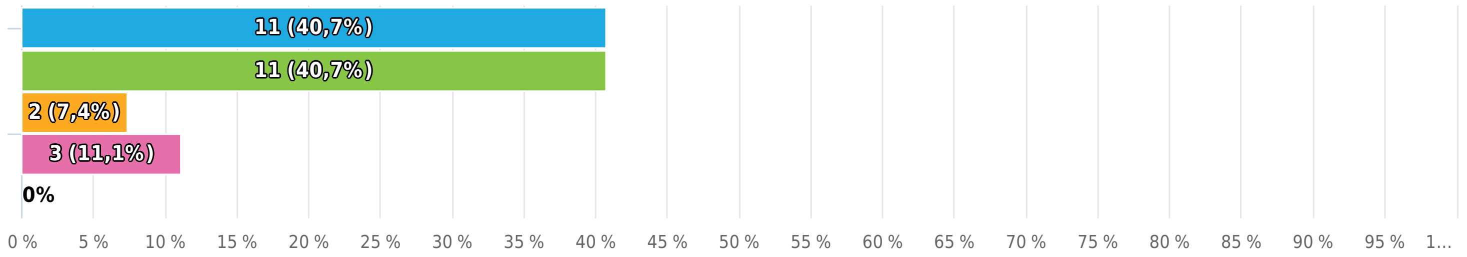

7 Byl jsi někdy léčen pro sekundární osteoporózu? Léky se jmenují bisfosfonáty, například Zometa, Zolendronát či Alendronát

Výběr z možností , zodpovězeno 27 x, nezodpovězeno 0 x

Možnosti odpovědí	Responzí	Podíl
Ano - v minulosti	1	3,7%
Ano - léčba stále v pravidelných intervalech probíhá	11	40,7%
Ne	15	55,6%

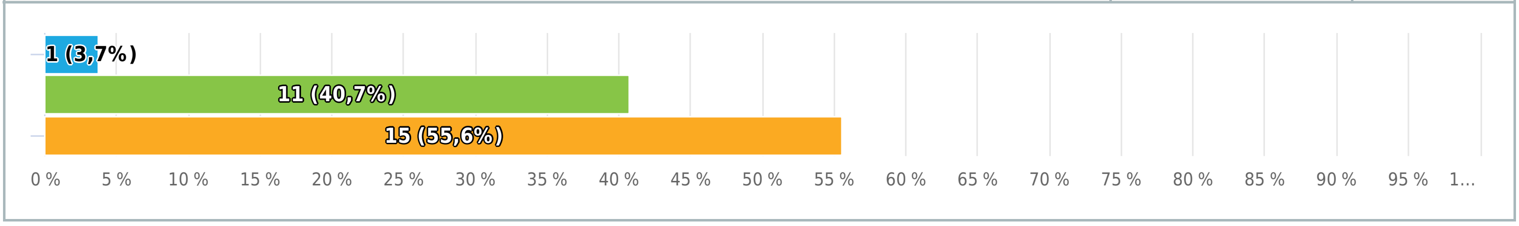

8 Zmírnily se obtíže charakteru bolesti zad po roce terapie?

Výběr z možností, více možných , zodpovězeno 12 x, nezodpovězeno 15 x

Možnosti odpovědí	Responzí	Podíl
Na bolesti zad jsem netrpěl ani před léčbou	2	16,7%
Ano, bolesti se zmírnily	7	58,3%
Ne, bolesti zad mám stále zhruba stejné	3	25,0%
Ne, bolesti zad se mi spíše zhoršily	0	0,0%

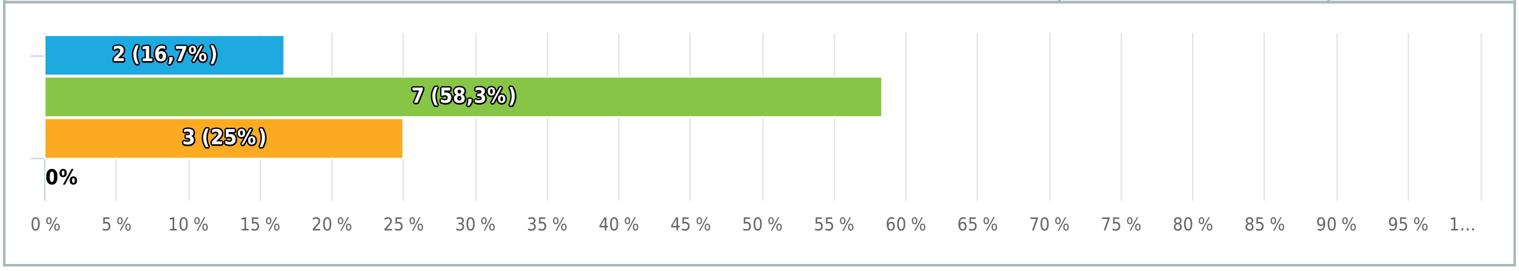

9 Zlepšilo se na terapii něco jiného?

Výběr z možností , zodpovězeno 12 x, nezodpovězeno 15 x

Možnosti odpovědí	Responzí	Podíl
Ne	11	91,7%
Ano - napiš, prosím, co konkrétně	1	8,3%

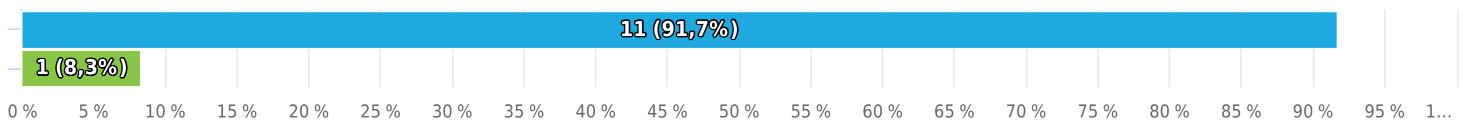

10 Měl jsi na terapii nějaké nepříjemné nežádoucí účinky?

Výběr z možností , zodpovězeno 10 x, nezodpovězeno 17 x

Možnosti odpovědí	Responzí	Podíl
Ne	1	10,0%
Ano, ale jen po první dávce	8	80,0%
Ano, pokaždé	1	10,0%

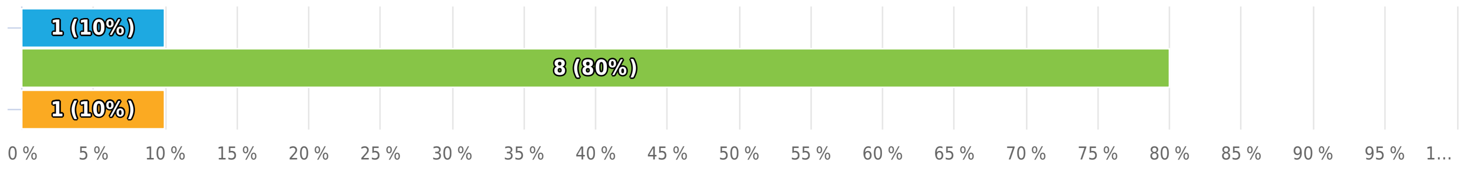

11 Na terapii mi přijde nejvíce zatěžující:

Výběr z možností, více možných , zodpovězeno 12 x, nezodpovězeno 15 x

Možnosti odpovědí	Responzí	Podíl
Terapie pro mě není výrazně zatěžující	5	41,7%
Způsob podání do žíly, nutnost zavedení kanyly a odběrů krve	3	25,0%
Nutnost být často v nemocnici	1	8,3%
Nutnost dojíždět do Prahy	2	16,7%
Horečka po podání	1	8,3%
Bolest	0	0,0%
Něco jiného: prosím popiš, co Tě ještě zatěžuje	3	25,0%

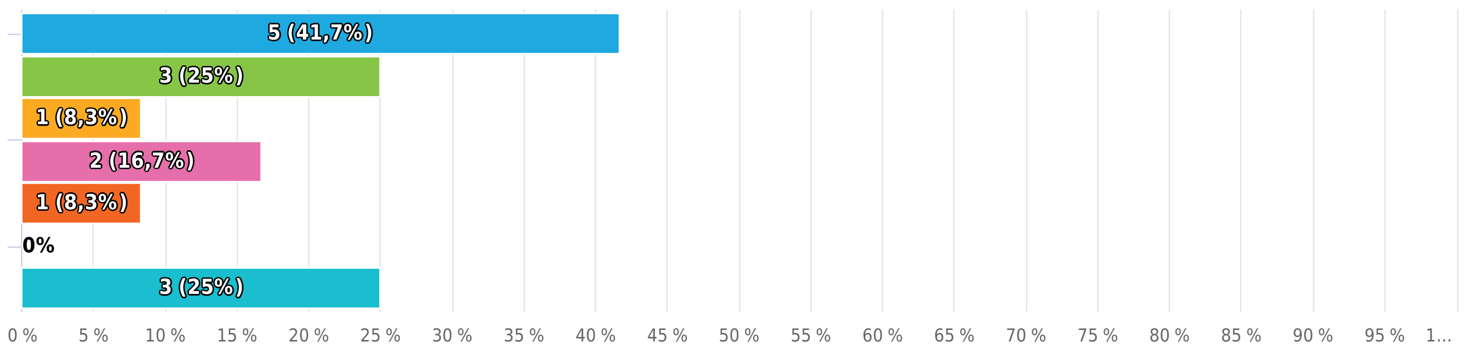

12 Kdybych se mohl rozhodnout zcela sám, chtěl bych tuto terapii dostávat?

Výběr z možností , zodpovězeno 12 x, nezodpovězeno 15 x

Možnosti odpovědí	Responzí	Podíl
Ano	5	41,7%
Ne	2	16,7%
Nevím	5	41,7%

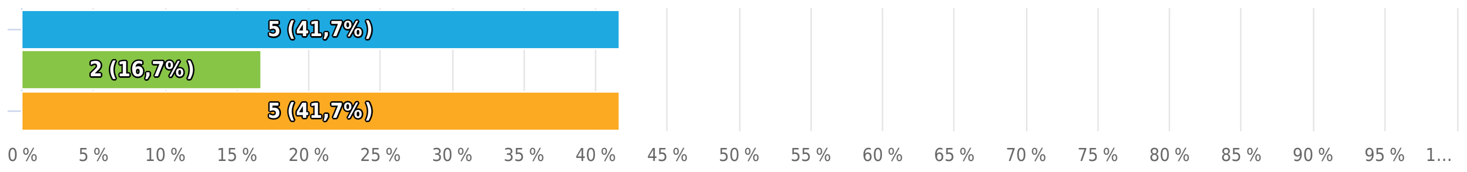

13 Máš skoliózu páteře?

Výběr z možností , zodpovězeno 27 x, nezodpovězeno 0 x

Možnosti odpovědí	Responzí	Podíl
Ano	7	25,9%
Ne	20	74,1%

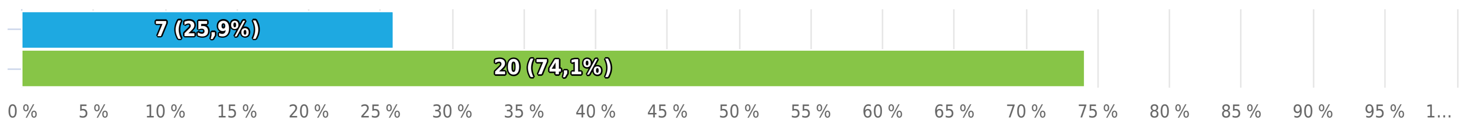

14 Pokud ano, vadí Ti skolióza páteře?

Výběr z možností , zodpovězeno 6 x, nezodpovězeno 21 x

Možnosti odpovědí	Responzí	Podíl
Vůbec nevadí	1	16,7%
Spíše nevadí	2	33,3%
Nevím	1	16,7%
Docela vadí	1	16,7%
Velice vadí	1	16,7%

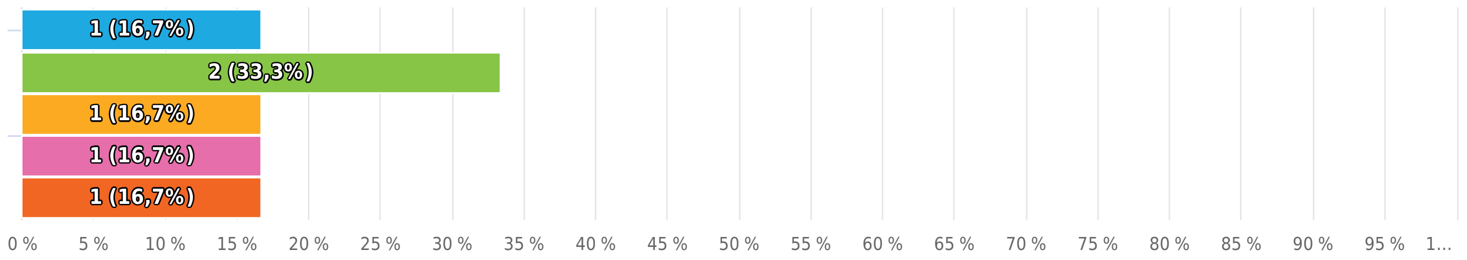

15 Co zejména je podle Tebe na skolióze nepříjemné? Můžeš vybrat jednu nebo i více odpovědí.

Výběr z možností, více možných , zodpovězeno 6 x, nezodpovězeno 21 x

Možnosti odpovědí	Responzí	Podíl
Bolest zad	4	66,7%
Obtíže se stabilizací v sedu	3	50,0%
Ztížené dýchání či příjem stravy	1	16,7%
To, jak vypadá	0	0,0%
Snížení celkové výšky	1	16,7%
Něco jiného: prosím, napiš, co to je	1	16,7%

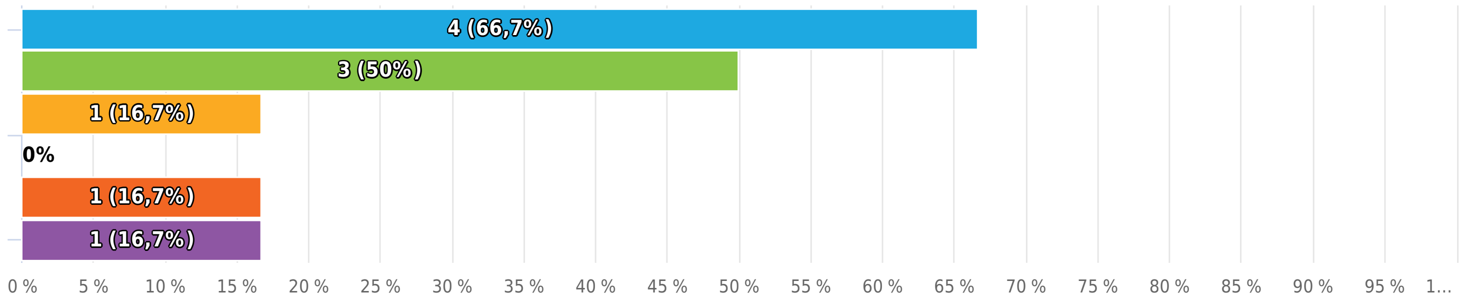

16 Chlapci s DMD mívají často menší výšku než jejich vrstevníci. Budeš-li výrazně menší než vrstevníci, pak Ti to:

Výběr z možností , zodpovězeno 27 x, nezodpovězeno 0 x

Možnosti odpovědí	Responzí	Podíl
Vůbec nevadí	5	18,5%
Spíše nevadí	7	25,9%
Nevím, nedokáži posoudit	8	29,6%
Docela vadí	6	22,2%
Velice vadí	1	3,7%

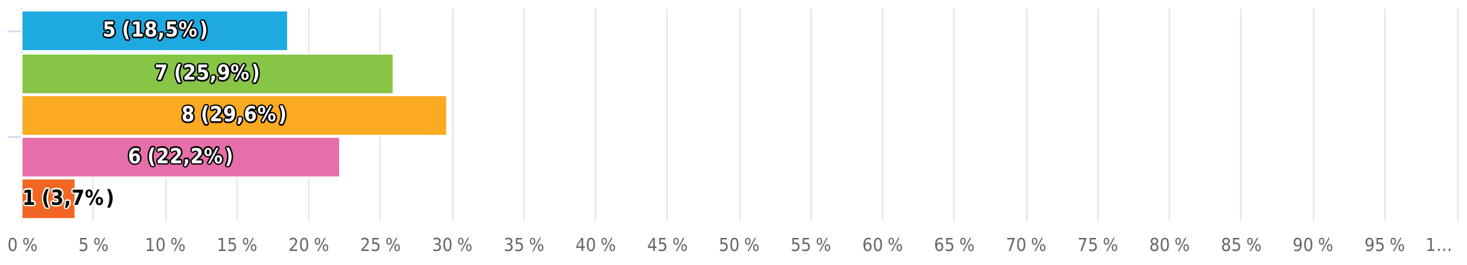

17 U chlapců s DMD se často setkáváme i s opožděním puberty. Pokud bys měl opožděnou pubertu, tedy dospíval navenek pomaleji než vrstevníci, pak Ti to:

Výběr z možností , zodpovězeno 27 x, nezodpovězeno 0 x

Možnosti odpovědí	Responzí	Podíl
Vůbec nevadí	6	22,2%
Spíše nevadí	5	18,5%
Nevím	11	40,7%
Docela vadí	4	14,8%
Velice vadí	1	3,7%

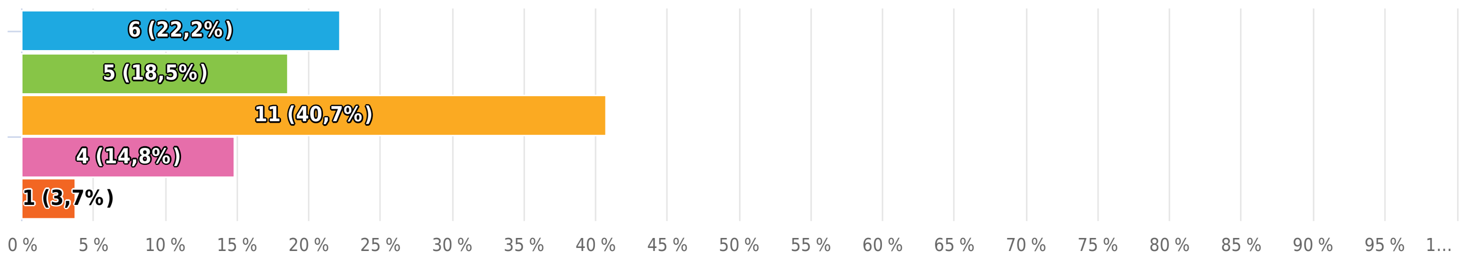

18 Napiš, co Ti na opožděné pubertě vadí (můžeš vybrat i více možností):

Výběr z možností, více možných , zodpovězeno 27 x, nezodpovězeno 0 x

Možnosti odpovědí	Responzí	Podíl
Nevadí mi	8	29,6%
Odlišný vzhled či jiné zájmy než mají vrstevníci	9	33,3%
Že Tě okolí vnímá více jako malého chlapce, snížení samostatnosti	10	37,0%
Riziko zdravotních komplikací – například nižší kostní densita (řidší kosti) v porovnání s chlapci, kteří mají pubertu dříve	5	18,5%
Strach, že si nenajdu partnerku / partnera nebo nezaložím vlastí rodinu	4	14,8%
Něco jiného: prosím napiš nám, co to je:	1	3,7%

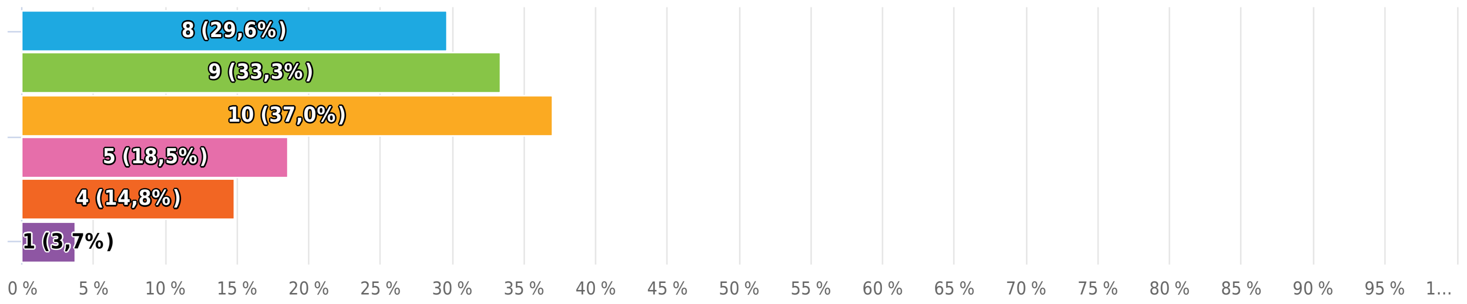

19 Přál by sis, aby se pubertální zrání u Tvého lékaře více sledovalo tak, aby Ti případně mohl včas doporučit konzultaci s odborníkem, který by mohl nabídnout léky k urychlení puberty?

Výběr z možností , zodpovězeno 27 x, nezodpovězeno 0 x

Možnosti odpovědí	Responzí	Podíl
Ano	6	22,2%
Ne	7	25,9%
Nevím	14	51,9%

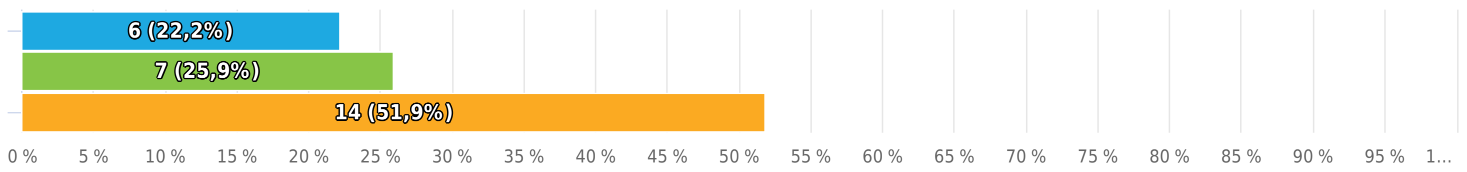

20 Jak moc by Ti vadilo pravidelně podstupovat v nervosvalové ambulanci takové vyšetření?

Výběr z možností , zodpovězeno 27 x, nezodpovězeno 0 x

Možnosti odpovědí	Responzí	Podíl
Vůbec nevadilo	4	14,8%
Spíše nevadilo	9	33,3%
Nevím	8	29,6%
Docela vadilo	5	18,5%
Velice vadilo	1	3,7%

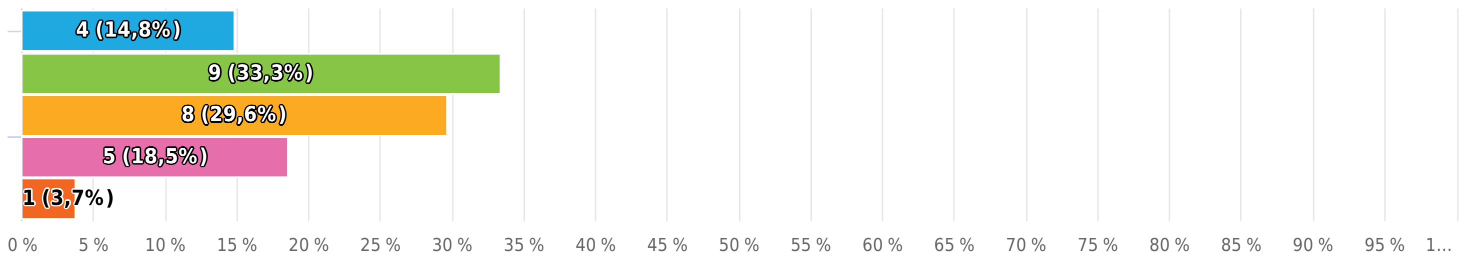

21 Seřaď prosím následující položky podle toho, jak jsou pro Tebe důležité

Seřazení dle důležitosti , zodpovězeno 18 x, nezodpovězeno 9 x

Odpověď	Důležitost
Být stejně velký jako vrstevníci	3,2
Co nejdéle si zachovat schopnost chodit a mít sílu v rukou potřebnou k běžným denním činnostem	0,0
Dospívat stejným tempem jako vrstevníci	0,0
Snížit co nejvíce riziko zlomenin	3,3
Být co nejvíce samostatný	4,0
Trávit co nejméně času v nemocnici	5,0

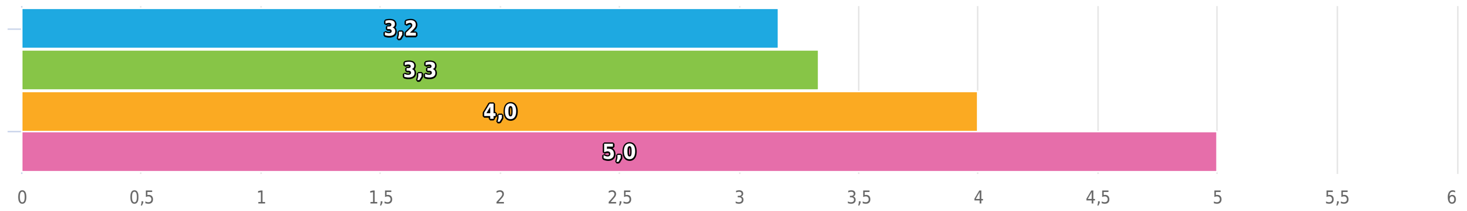

22 Byl jsi někdy léčen pro opožděnou pubertu?

Výběr z možností , zodpovězeno 27 x, nezodpovězeno 0 x

Možnosti odpovědí	Responzí	Podíl
Ano - léčba stále probíhá	5	18,5%
Ano - léčba již byla ukončena	0	0,0%
Ne	22	81,5%

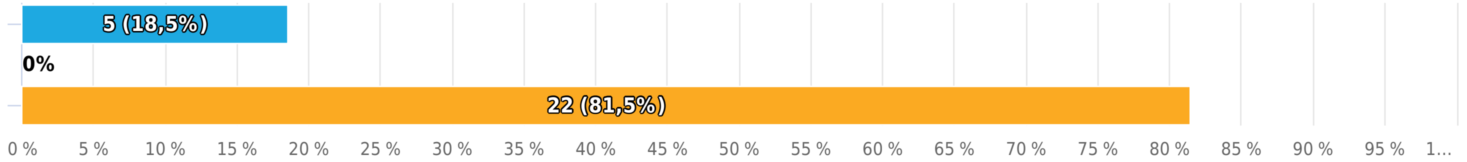

23 Pokud u tebe léčba proběhla, jak jsi s jejím efektem spokojený?

Výběr z možností , zodpovězeno 4 x, nezodpovězeno 23 x

Možnosti odpovědí	Responzí	Podíl
Velice spokojený	1	25,0%
Docela spokojený	1	25,0%
Nevím	2	50,0%
Spíše nespokojený	0	0,0%
Velice nespokojený	0	0,0%

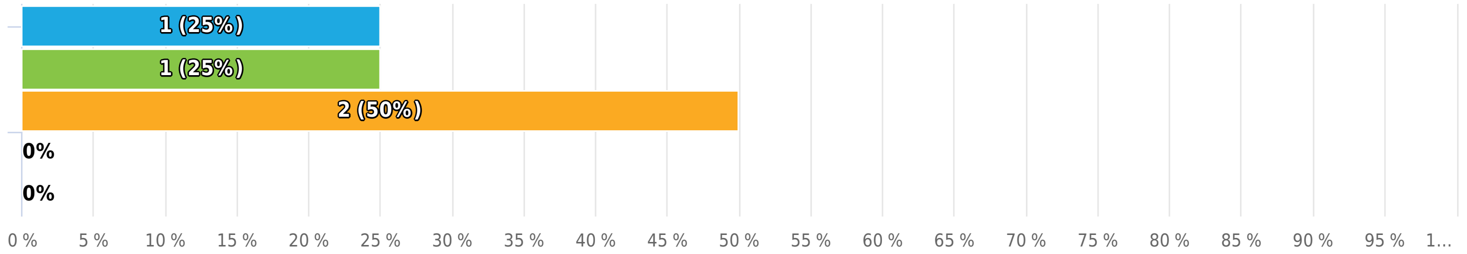

24 Vyber, v čem ti léčba opožděné puberty pomohla (můžeš zvolit více možností)

Výběr z možností, více možných , zodpovězeno 5 x, nezodpovězeno 22 x

Možnosti odpovědí	Responzí	Podíl
Zlepšení svalové síly	0	0,0%
Jsem vyšší než jsem byl předtím	0	0,0%
Jsem spokojenější se svým vzhledem	1	20,0%
Mám lepší náladu, více energie	2	40,0%
Jsem samostatnější	0	0,0%
Žádné zlepšení nepozoruji	0	0,0%
Jiná odpověď - prosím, napiš:	2	40,0%

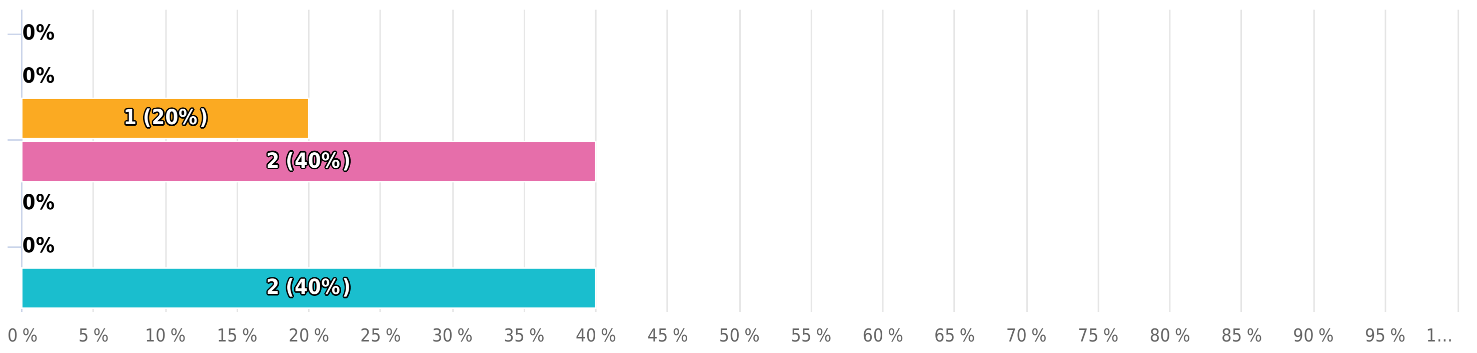

25 Který z následujících možných efektů léčby opožděné puberty je pro Tebe osobně nejdůležitější a který nejméně? Seřaď od 1 (nejdůležitjší) do 5 (nejméně důležitý).

Seřazení dle důležitosti , zodpovězeno 17 x, nezodpovězeno 10 x

Odpověď	Důležitost
Zlepšení svalové síly	1,6
Urychlení růstu, vyšší dosažená výška v dospělosti	0,0
Dospělejší vzhled (růst vousů apod.)	3,3
Zlepšení kostního zdraví, snížení rizika vzniku zlomenin	0,0
Zlepšení nálady	3,2

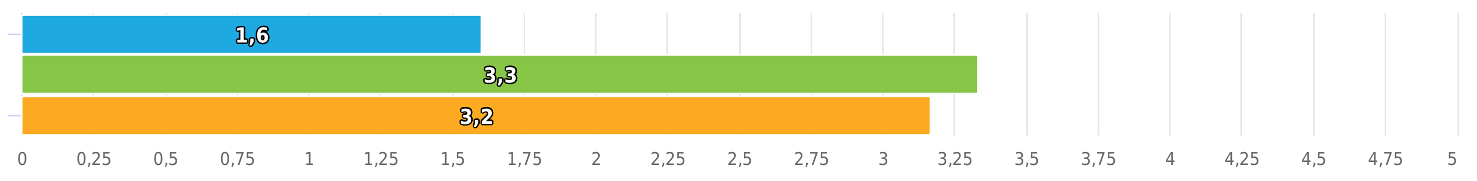

26 Kdyby ses mohl rozhodnout sám, chtěl bys umělé spuštění puberty podstoupit, pokud by u Tebe nenastalo dospívání samovolně?

Výběr z možností , zodpovězeno 27 x, nezodpovězeno 0 x

Možnosti odpovědí	Responzí	Podíl
Ano	6	22,2%
Ne	6	22,2%
Nevím	15	55,6%

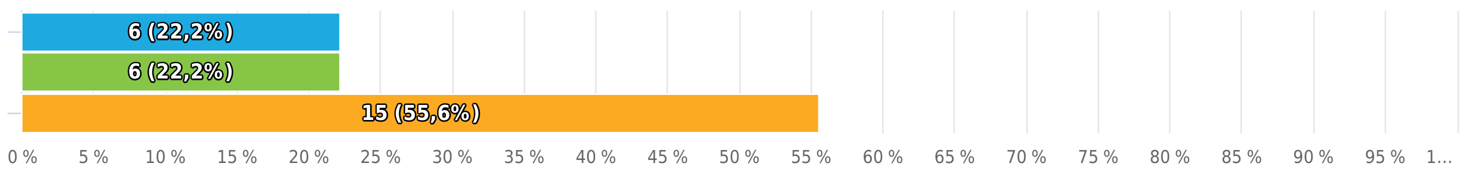

27 Kdybys měl z následujících endokrinologických komplikací vybrat jednu, která Ti vadí nejvíce, která by to byla?

Výběr z možností , zodpovězeno 27 x, nezodpovězeno 0 x

Možnosti odpovědí	Responzí	Podíl
Opožděná puberta	2	7,4%
Malá výška	6	22,2%
Osteoporóza (křehké kosti, vyšší riziko zlomenin)	19	70,4%

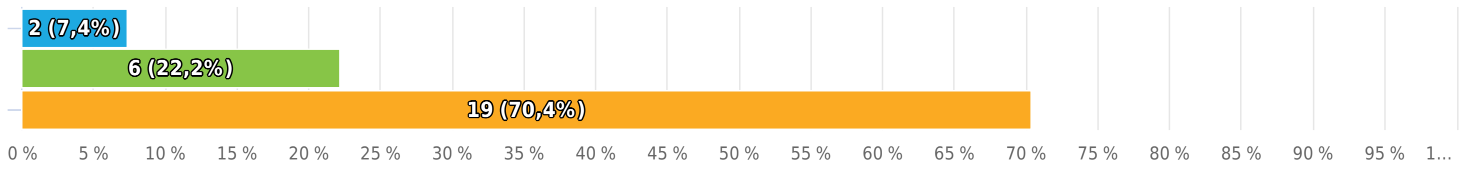

28 Máš na nás nějaké připomínky nebo bys chtěl něco doplnit?

Textová odpověď, zodpovězeno 27 x, nezodpovězeno 0 x

- -
- Asi ne
- dík
- Chtěl bych být jako ostatní
- Matýsek ještě naštěstí tyto věci příliš neřeší
- (2x) ne
- (8x) Ne
- Ne
- nemám
- (2x) Nemám
- Neumím se vžít do pocitů budoucího dospívajícího syna.
- nevím
- Nic
- Nic nechci doplnit.
- Přál bych co nejdřív abych bila léčba na moji diagnózu
- Rad jsem vyplnil s maminkou
- Zatím ne
- zeptejte se na každodenní činnosti. například jídlo a pití, osobní hygiena. zeptejte se na náladu pacienta.

Nastavení dotazníku

Povolit odeslat vícekrát?	✓
Povolit návrat k předchozím otázkám?	✓
Zobrazovat čísla otázek?	✓
Oznámení o vyplnění dotazníku na e-mail?	✓
Ochrana heslem?	
IP omezení?	

Příloha: dotazník

Sledování endokrinologických komplikací a léčby u chlapců s DMD

1 Jaké jsou Tvoje iniciály? (Napiš prosím první písmeno ze svého jména a ze svého příjmení)

Nápověda k otázce: *(slouží pro přiřazení stejného identifikátoru, jaký mají i data o laboratorních testech v naší databázi tak, abychom je mohli správně přiřadit k sobě. Při zpracování dat nebude již nikde uvedeno)*

2 Kolik je ti let?

3 Kdo odpovídá na otázky dotazníku?

Nápověda k otázce: *Vyberte jednu odpověď*

○ Já sám za sebe (s případnou technickou dopomocí) ○ Vyplňuje za mě rodič / opatrovník

4 Užíváš kortikoidy?

Nápověda k otázce: *Vyberte jednu odpověď*

○ Ano ○ Ne

Následující otázky se budou týkat zlomenin a případné léčby osteoporózy.

5 Měl jsi někdy nějakou zlomeninu?

Nápověda k otázce: *Vyberte jednu nebo více odpovědí*

☐ Ano - ruky nebo nohy ☐ Ano - kompresivní zlomeninu obratlů (lékař to poznal dle RTG páteře) ☐ Ano - jinou ☐ Ne, nikdy jsem nic zlomeného neměl.

6 Trpíš na bolesti zad?

Nápověda k otázce: *Vyberte jednu odpověď*

◯ Ne nebo jen výjimečně ◯ Občas, bolest je mírná ◯ Občas, bolest je silná ◯ Velmi často ◯ Ano, každý den

7 Byl jsi někdy léčen pro sekundární osteoporózu? Léky se jmenují bisfosfonáty, například Zometa, Zolendronát či Alendronát

Nápověda k otázce: *Vyberte jednu odpověď*

◯ Ano - v minulosti ◯ Ano - léčba stále v pravidelných intervalech probíhá ◯ Ne

Následující otázky (8-12) jsou jen pro chlapce, kteří užívají bisfosfonáty 1 rok a déle. Pokud je to Tvůj případ, prosím, odpověz na ně. Pokud bisfosfonáty neužíváš, můžeš otázky přeskočit.

8 Zmírnily se obtíže charakteru bolesti zad po roce terapie?

Nápověda k otázce: *Vyberte jednu odpověď*

☐ Na bolesti zad jsem netrpěl ani před léčbou ☐ Ano, bolesti se zmírnily ☐ Ne, bolesti zad mám stále zhruba stejné ☐ Ne, bolesti zad se mi spíše zhoršily

9 Zlepšilo se na terapii něco jiného?

Nápověda k otázce: *Vyberte jednu odpověď*

◯ Ne

◯ Ano - napiš, prosím, co konkrétně ____________

10 Měl jsi na terapii nějaké nepříjemné nežádoucí účinky?

Nápověda k otázce: *Vyberte jednu odpověď*

◯ Ne ◯ Ano, ale jen po první dávce ◯ Ano, pokaždé

11 Na terapii mi přijde nejvíce zatěžující:

Nápověda k otázce: *Vyberte jednu nebo více odpovědí*

- ☐ Terapie pro mě není výrazně zatěžující
- ☐ Způsob podání do žíly, nutnost zavedení kanyly a odběrů krve
- ☐ Nutnost být často v nemocnici
- ☐ Nutnost dojíždět do Prahy
- ☐ Horečka po podání
- ☐ Bolest
- ☐ Něco jiného: prosím popiš, co Tě ještě zatěžuje ________

12 Kdybych se mohl rozhodnout zcela sám, chtěl bych tuto terapii dostávat?

Nápověda k otázce: *Vyberte jednu odpověď*

○ Ano ○ Ne ○ Nevím

Následující otázky se týkají skoliózy páteře, tedy jejího chybného zakřivení, které pozná lékař z klinického vyšetření v ambulanci nebo z rentgenového snímku páteře.

13 Máš skoliózu páteře?

Nápověda k otázce: *Vyberte jednu odpověď*

○ Ano ○ Ne

14 Pokud ano, vadí Ti skolióza páteře?

Nápověda k otázce: *Vyberte jednu odpověď*

○ Vůbec nevadí ○ Spíše nevadí ○ Nevím ○ Docela vadí ○ Velice vadí

15 Co zejména je podle Tebe na skolióze nepříjemné? Můžeš vybrat jednu nebo i více odpovědí.

Nápověda k otázce: *Vyberte jednu nebo více odpovědí*

- ☐ Bolest zad
- ☐ Obtíže se stabilizací v sedu
- ☐ Ztížené dýchání či příjem stravy
- ☐ To, jak vypadá
- ☐ Snížení celkové výšky
- ☐ Něco jiného: prosím, napiš, co to je ________

Následující otázky se budou týkat růstu a puberty.

16 Chlapci s DMD mívají často menší výšku než jejich vrstevníci. Budeš-li výrazně menší než vrstevníci, pak Ti to:

Nápověda k otázce: *Vyberte jednu odpověď*

- ◯ Vůbec nevadí
- ◯ Spíše nevadí
- ◯ Nevím, nedokáži posoudit
- ◯ Docela vadí
- ◯ Velice vadí

17 U chlapců s DMD se často setkáváme i s opožděním puberty. Pokud bys měl opožděnou pubertu, tedy dospíval navenek pomaleji než vrstevníci, pak Ti to:

Nápověda k otázce: *Vyberte jednu odpověď*

- ◯ Vůbec nevadí
- ◯ Spíše nevadí
- ◯ Nevím
- ◯ Docela vadí
- ◯ Velice vadí

18 Napiš, co Ti na opožděné pubertě vadí (můžeš vybrat i více možností):

Nápověda k otázce: *Vyberte jednu nebo více odpovědí*

- ☐ Nevadí mi
- ☐ Odlišný vzhled či jiné zájmy než mají vrstevníci
- ☐ Že Tě okolí vnímá více jako malého chlapce, snížení samostatnosti
- ☐ Riziko zdravotních komplikací – například nižší kostní densita (řidší kosti) v porovnání s chlapci, kteří mají pubertu dříve
- ☐ Strach, že si nenajdu partnerku / partnera nebo nezaložím vlastí rodinu
- ☐ Něco jiného: prosím napiš nám, co to je: ____________

19 Přál by sis, aby se pubertální zrání u Tvého lékaře více sledovalo tak, aby Ti případně mohl včas doporučit konzultaci s odborníkem, který by mohl nabídnout léky k urychlení puberty?

Nápověda k otázce: *Vyberte jednu odpověď*

- ◯ Ano
- ◯ Ne
- ◯ Nevím

Toto vyšetření obnáší i vyšetření genitálií – lékař se při tom podívá a ohodnotí pohledem vyzrálost (dle speciální stupnice dle Tannera) a dále změří objem varlat pomocí orchidometru – vypadá jako barevné různě veliké korálky, které se přiloží k šourku a poměří s objemem varlat. Dále bývá nutný odběr krve k vyšetření pohlavních hormonů (nyní již součástí našeho běžného screeningu). Dle toho lékař pozná, jak daleko je Tvé pubertální zrání a pokud puberta nenastoupí do 14 let, může doporučit její vyvolání pomocí léků.

20 Jak moc by Ti vadilo pravidelně podstupovat v nervosvalové ambulanci takové vyšetření?

Nápověda k otázce: *Vyberte jednu odpověď*

○ Vůbec nevadilo ○ Spíše nevadilo ○ Nevím ○ Docela vadilo ○ Velice vadilo

21 Seřaď prosím následující položky podle toho, jak jsou pro Tebe důležité

Nápověda k otázce: *Změň pořadí položek tak, že je přetáhneš myší: 1 - nejvíce důležitá, 6 - nejméně důležitá*

Být stejně velký jako vrstevníci	
Co nejdéle si zachovat schopnost chodit a mít sílu v rukou potřebnou k běžným denním činnostem	
Dospívat stejným tempem jako vrstevníci	
Snížit co nejvíce riziko zlomenin	
Být co nejvíce samostatný	
Trávit co nejméně času v nemocnici	

Následující otázky se týkají léčby opožděné puberty. Otázky 23-24 se týkají jen chlapců, u kterých proběhla či probíhá léčba pro opožděnou pubertu.

22 Byl jsi někdy léčen pro opožděnou pubertu?

Nápověda k otázce: *Vyberte jednu odpověď*

○ Ano - léčba stále probíhá ○ Ano - léčba již byla ukončena ○ Ne

23 Pokud u tebe léčba proběhla, jak jsi s jejím efektem spokojený?

Nápověda k otázce: *Vyberte jednu odpověď*

○ Velice spokojený ○ Docela spokojený ○ Nevím ○ Spíše nespokojený ○ Velice nespokojený

24 Vyber, v čem ti léčba opožděné puberty pomohla (můžeš zvolit více možností)

Nápověda k otázce: *Vyberte jednu nebo více odpovědí*

- ☐ Zlepšení svalové síly
- ☐ Jsem vyšší než jsem byl předtím
- ☐ Jsem spokojenější se svým vzhledem
- ☐ Mám lepší náladu, více energie
- ☐ Jsem samostatnější
- ☐ Žádné zlepšení nepozoruji
- ☐ Jiná odpověď - prosím, napiš: ________

25 Který z následujících možných efektů léčby opožděné puberty je pro Tebe osobně nejdůležitější a který nejméně? Seřaď od 1 (nejdůležitjší) do 5 (nejméně důležitý).

Nápověda k otázce: *Změň pořadí položek tak, že je přetáhneš myší (1. - nejdůležitější, 5. - nejméně důležitá)*

Zlepšení svalové síly	
Urychlení růstu, vyšší dosažená výška v dospělosti	
Dospělejší vzhled (růst vousů apod.)	
Zlepšení kostního zdraví, snížení rizika vzniku zlomenin	
Zlepšení nálady	

26 Kdyby ses mohl rozhodnout sám, chtěl bys umělé spuštění puberty podstoupit, pokud by u Tebe nenastalo dospívání samovolně?

Nápověda k otázce: *Vyberte jednu odpověď*

- ◯ Ano
- ◯ Ne
- ◯ Nevím

27 Kdybys měl z následujících endokrinologických komplikací vybrat jednu, která Ti vadí nejvíce, která by to byla?

Nápověda k otázce: *Vyberte jednu odpověď*

- ◯ Opožděná puberta
- ◯ Malá výška
- ◯ Osteoporóza (křehké kosti, vyšší riziko zlomenin)

28 Máš na nás nějaké připomínky nebo bys chtěl něco doplnit?

Hotovo. :-) Velice děkujeme za Tvůj čas a za vyplnění tohoto dotazníku!
